# Dual-Arm Coordinated Control Strategy Based on Modified Sliding Mode Impedance Controller

**DOI:** 10.3390/s21144653

**Published:** 2021-07-07

**Authors:** Xuefei Liu, Xiangrong Xu, Zuojun Zhu, Yanglin Jiang

**Affiliations:** School of Mechanical Engineering, Anhui University of Technology, Ma’anshan 243032, China; liuxf618@163.com (X.L.); mrzero1956@163.com (Z.Z.); jiangyl96@163.com (Y.J.)

**Keywords:** dual-arm robots, sliding mode control, impedance control, coordinated operation, target object

## Abstract

To meet the high-accuracy position/force control requirements of dual-arm robots for handling a target object, a control algorithm for dual-arm robots based on the modified sliding mode impedance controller MSMIC(tanh) is proposed. First, the combinative kinematics equation of the dual-arm robots and the unified dynamics model combining the manipulated object is established. Second, according to the impedance control motion model for the object, the desired joint angular accelerations of the manipulators are obtained, and the sliding mode controller based on the hyperbolic tangent function as the switch function is introduced to design the coordinated control strategy for dual-arm robots. The stability and convergence of the designed controller are proved according to the Lyapunov function theory. Finally, the operation tasks of the coordinated transport the target object for dual-arm robots are carried out in the simulated experiment environment. Simulation results show that the proposed control scheme can stably output the required internal force and achieve a high-precision trajectory tracking effect while reducing the periodic torque and joint chattering amplitude generated in the conventional sliding mode control algorithm.

## 1. Introduction

At present, robots are used and studied in many different fields; examples include automobile manufacturing, space manipulators, underwater exploration tools, surgical operations, and medical assistance robots. In recent years, with the transformation of manufacturing tasks, the drawbacks of traditional manipulators, such as single operation, low payload capacity, and inability to coordinate the operation, etc., have become increasingly obvious [1]. Dual-arm robots have drawn the increased attention of numerous researchers because of their cooperative and mutual assistance advantages. Among the research on many significant technologies, the compliant control method and the coordinated handling the target object of dual-arm robots has become the main research direction [2,3,4]. Therefore, many researchers at home and abroad have carried out extensive ongoing research and exploration for dual-arm cooperative manipulators.

When dual-arm robot systems are applied to grasp the object, it needs to control both the motion trajectory and the internal force for the manipulated object [5]. According to whether the robot end-effector is in contact with the external environment, the tasks performed by the robot can be divided into two categories. The first category is noncontact task; the robot is free to track the desired trajectory in the structured environment. For this type of task, the pure position control algorithm can be used to complete the desired task. The second type is the contact task, such as coordinated handling, surface polishing, automobile polishing, etc. The fine position deviation of the robot end-effector may lead to enormous contact force on object surface, so it is necessary to add force control part in the controller to improve the effective operation accuracy of the end-effector [6,7,8]. For the robot with contact force, a compliant control strategy is needed. The impedance control method first was proposed by Hogan to solve the problem of contact force encountered by the robot when performing the task and presented a new control way to realize the smooth motion for the held object when it was disturbed by the external environment [9,10]. Impedance control is to transform the real-time force error signals measured by the force/torque sensors mounted on the joints into position error signals. The exact force control is achieved by calculating the real-time position error. It is a force control method with stable and excellent control performance [11,12].

There have been many different impedance control approaches to the robot’s interaction operation system. Koga and Wimböck [13,14] proposed two different impedance control ways for the coordinated robot system; they further put the interaction control between the target object and the external environment. Schneider et al. [15] enlarged creatively the construction of the impedance control algorithm for a dual-arm robot system. The object impedance other than just the impedance of robots’ end-effectors is achieved through their method. Afterward, the internal and external object impedance were successfully combined by Caccavale [16], whose algorithm can simultaneously eliminate the dependence of the manipulated object dynamics by proper impedance control parameters.

To maintain safe robot–environment interaction, the hybrid position/force control scheme is another widely adopted for interactive control system in dual-arm cooperative robots. This control idea is that the robots employ a force control strategy in the constrained subspace and position control strategy in the free subspace [17]. Re et al. proposed an adaptive hybrid position/force control algorithm with closed-chain kinematics and uncertain dynamics, which is a relatively advanced hybrid position/force control technique [6]. However, this control method needs to switch position/force control mode in the transition process of two subspaces, which cannot guarantee compliance performance of the robot control system.

The dynamics modeling of multi-degree-of-freedom (DOF) dual-arm robots is more complicated than that of traditional single manipulators. The established dynamics equation is only an approximation of the real model system, so the model must contain uncertain factors, such as parameter uncertainties, external environment disturbances, and frictions between joints. Designing controller with strong adaptability, high nonlinearity, and superior control performance is an enormous challenge for researchers [18,19]. In actual industry applications, we need a controller that can overcome modeling uncertainties and robot end-effector time-varying disturbances [20].

Sliding mode controller has the advantages of strong adaptive ability, robustness, generalization ability, portability, and simple physical implementation [21,22,23]. The sliding mode control way has been widely used in robot control systems with the parameter uncertainties and external disturbances [23,24]. Maolin Jin [25] proposed a terminal sliding mode control to the time delay estimation to compensate for the model parameter errors of the manipulators, which improved the robustness and accuracy of the control system. However, the design of the sliding mode controller exists a fierce torque chattering, which makes it difficult to complete the dual-arm cooperation tasks. In recent years, the research of adaptive fuzzy sliding mode controller using the universal approximation theorem to compensate the uncertain terms in the dynamics model has achieved good control results. Yuguang Zhou [26] combined the Lagrange dynamics equation with an adaptive fuzzy sliding mode controller; the tested simulation results show that the designed controller can effectively resist the disturbances of the external environment and achieve high-precision trajectory tracking effect and small chattering amplitude of the joint control torques.

From the aforementioned study, it is notable that the impedance control algorithm in the Cartesian space is a better compliant control strategy in that it can transform force error signals into position error signals [27]. Impedance control can achieve better force tracking results [28,29]. However, pure impedance control has a general trajectory tracking accuracy and cannot get a high-precision trajectory/force tracking effect for dual-arm cooperative manipulators. In this article, a modified sliding mode impedance controller MSMIC(tanh) is proposed to meet the requirements of precise position/force control for a dual-arm robots to handle a target object. On the basis of impedance control and sliding mode control, the universal approximation property for sliding mode function is used to achieve the high-accuracy control requirements of the target object. At the same time, the hyperbolic tangent function instead of the traditional signum function can reduce the chattering amplitude of the control output torques and joint angles.

The stability and convergence of the designed controller are proved by the Lyapunov function. The dual-arm simulation system is built-in MATLAB/SIMULINK, and the real-time signals measured by the force/torque sensors mounted on the joints and the position/velocity sensors mounted on the end-effectors are used as feedback information to realize the feedback control. The controller proposed can output the required contact internal force stably and get high-precision trajectory tracking results while reducing the periodic torque and joint chattering extent generated in the traditional sliding mode controller.

The rest of the article is outlined as follows. In Section 2, we describe the combinative kinematics model of the dual-arm robots. In Section 3, we establish the unified dynamics equation between the dual-arm robots and the object. In Section 4, we present the design of a modified sliding mode impedance controller based on the dual-arm coordinated operation system. In Section 5, we present the results of the finished series of simulation experiments. In Section 6, we discuss the research content and application direction of this paper. The conclusion is presented in the final section.

## 2. Combinative Kinematics Model of the Dual-Arm Robots

Different from the serial manipulators of the traditional multiple degrees of freedom, the coordinated system of grasping the target object for the dual-arm robots is more complex. Establishing the combinative kinematics model about the dual-arm is the basis for realizing the motion planning control and compliant coordinated operation.

**Figure 1:**
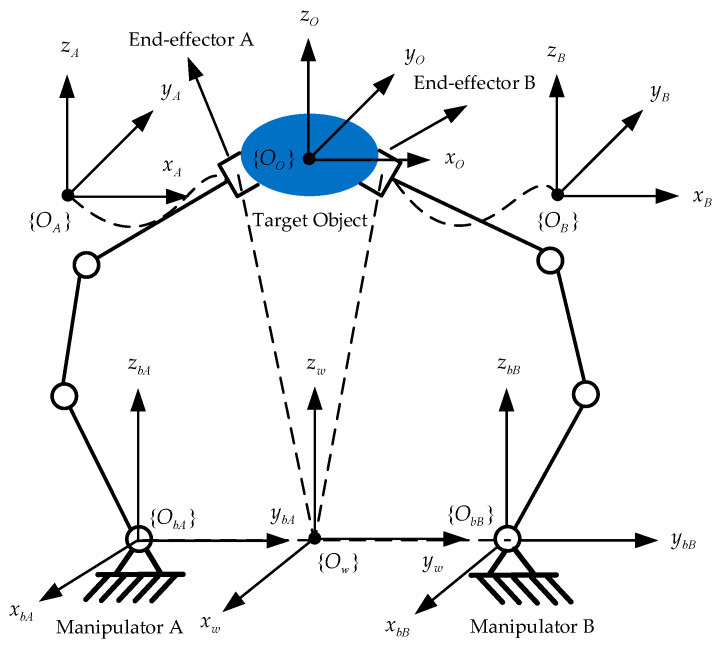
The diagram of the coordinate system of the dual-arm robots.

Aiming at handling the object for the end-effectors of the dual-arm cooperative manipulators, the diagram of the coordinate system of the dual-arm robots is shown in Figure 1. The meaning of each coordinate system in the Figure 1 is explained as the following: $\left\{O_{w}\right\}$ is the world coordinate system of the entire closed chain constraint structure, $\left\{O_{bA}\right\}$ and $\left\{O_{bB}\right\}$ are the base coordinate system of the robot A and robot B, respectively, $\left\{O_{A}\right\}$ and $\left\{O_{B}\right\}$ represent the end-effector coordinate system of the robot A and robot B, respectively, and $\left\{O_{O}\right\}$ denotes the center of mass (COM) reference coordinate system of the target object [30].

The pose constraint relationship between the dual-arm robots end-effectors, the fixed bases, the target object, and the world coordinate system is shown as the following two equations:

(1) {Tow=TbAw⋅TAbA⋅ToATow=TbBw⋅TBbB⋅ToB

where Tow is the pose transformation matrix of the object with its origin in the object’s COM with regard to the world coordinate system, TbAw and TbBw are the pose transformation matrix of the base coordinate systems of the robot A and robot B with regard to the world coordinate system, respectively, TAbA and TBbB denote the pose transformation matrix of the end-effectors with regard to the base coordinate system, respectively, ToA and ToB represent the pose transformation matrix of the object’s COM with regard to the end-effector coordinate systems of the dual-arm robots, respectively. Then, the forward kinematics equations for the manipulators can be obtained:

(2) {TAbA=TwbA⋅Tow⋅TAoTBbB=TwbB⋅Tow⋅TBo

The expected kinematics characteristics of the rigid object should be obtained in advance for the dual-arm robots’ coordinated operation transport, which can be determined by a homogeneous transformation Tow. The other pose transformation matrices are all constant matrices, so that the expressions TAbA and TBbB can be easily calculated. Then, according to the homogeneous matrices TAbA and TBbB, the pose transformation matrix TAw and TBw of the dual-arm end-effectors with regard to the world frame can be characterized using the following equations:

(3) {TAw=TbAw⋅TAbATAw=TbBw⋅TBbB

Suppose that the dual-arm system is encompassed two *n*-DOF serial manipulators with the same space structures. For manipulator A, the matrix TAw is expressed as follows:

(4) TAw=[RApA00]

where $R_{A}\in R^{3\times 3}$ and $p_{A}\in R^{3\times 1}$ are a rotation matrix and a position vector for the manipulator A, respectively. The orientation vector $\omega_{A}\in R^{3\times 1}$ can be calculated by this rotation matrix and the end-effector pose for the manipulator A can be described by using the vector $x_{A}={\left[p_{A}^{T},\omega_{A}^{T}\right]}^{T}\in R^{6\times 1}$. Similarly, this vector $x_{B}={\left[p_{B}^{T},\omega_{B}^{T}\right]}^{T}\in R^{6\times 1}$ depicted with the manipulator B can be obtained; then, $x={\left[x_{A}^{T},x_{B}^{T}\right]}^{T}\in R^{12\times 1}$.

Set $q={\left[q_{A}^{T},q_{B}^{T}\right]}^{T}\in R^{2n\times 1}$ as the joint position vector of the dual-arm robots. The angular velocity $\dot{q}$ in the joint space can be mapped to the linear velocity $\dot{x}$ in the Cartesian space by the Jacobian matrix J(q). Through the kinematics analysis for the dual-arm robots, the following relation is satisfied:

(5) $$\dot{x}=J(q)\dot{q}$$

Taking the derivative on both sides of Equation (5) with respect to time *t*, we can obtain:

(6) $$\ddot{x}=J(q)\ddot{q}+\dot{J}(q)\dot{q}$$

Then, the joint acceleration control vector of the dual-arm robots can be expressed as:

(7) $$\ddot{q}=J^{-1}(q)(\ddot{x}-\dot{J}(q)\dot{q})$$

## 3. Unified Dynamics Modeling of Dual-Arm Robots and Target Object

### 3.1. Combinative Dynamics Modeling

To realize the coordinated handling and compliant control of the target object for the dual-arm cooperative robots, the joint space combinative dynamics equation of the dual-arm can be written in compact form as:

(8) $$M(q)\ddot{q}+C(q,\dot{q})\dot{q}+G(q)+\tau_{end}+\tau_{d}=\tau$$

where $M(q)=\mathrm{blockdiag}(M_{A}(q_{A}),M_{B}(q_{B}))\in R^{2n\times 2n}$ is the combinative symmetric positive definite inertia matrix of the dual-arm robots, $C=\mathrm{blockdiag}(C_{A},C_{B})\in R^{2n\times 2n}$ is the combinative Coriolis and centrifugal force matrix, $G={\left[G_{A}^{T},G_{B}^{T}\right]}^{T}\in R^{2n\times 1}$ is the combinative gravitational force vector, $\tau_{end}={\left[\tau_{endA}^{T},\tau_{endB}^{T}\right]}^{T}\in R^{2n\times 1}$ and $\tau_{d}={\left[\tau_{dA}^{T},\tau_{dB}^{T}\right]}^{T}\in R^{2n\times 1}$ represent the equivalent torque of the end-effectors when contacting with the target object and the disturbance torque of the external environment acting on the robot joints, respectively, and $\tau ={\left[\tau_{A}^{T},\tau_{B}^{T}\right]}^{T}\in R^{2n\times 1}$ is the control torque in joint space of robots.

To obtain a unified dynamics model of the dual-arm robots combined with the object, the joint space dynamics equation needs to be transformed into the task space dynamics equation. Suppose the generalized driving force at the end-effectors is F, and the following Equation (9) can be obtained according to the virtual work principle [31,32] (For redundant manipulator, the Jacobian pseudoinverse matrix $J^{+}(q)$ is used to replace the inverse matrix $J^{-1}(q)$):

(9) $$F=J^{-T}(q)\tau$$

where $J^{-T}(q)$ is transpose of the inverse Jacobian matrix $J^{-1}(q)$.

Considering the uncertainties of dynamics modeling (frictions between joints, the errors between the ideal model parameters and the actual model parameters, etc.) and combining Equation (5) with Equations (7)–(9), the combinative dynamics equation of the dual-arm robots in the Cartesian space is established:

(10) $$M_{X}(q)\ddot{x}+C_{X}(q,\dot{q})\dot{x}+G_{X}(q)+F_{end}+F_{d}+\Phi (q,\dot{q},\ddot{q})=F$$

where $M_{X}(q)$, $C_{X}(q,\dot{q})$, and $G_{X}(q)$ are the combinative inertia matrix of the dual-arm robots in the Cartesian space, Coriolis and centrifugal force matrix, and gravitational force vector, respectively, $F_{end}$ and $F_{d}$ denote the reaction force generated by the operation force at the end-effectors and the disturbance force acted on the manipulator by the external environment, respectively, $\Phi (q,\dot{q},\ddot{q})$ is the uncertainty term of the robot model parameters, and $||\Phi (q,\dot{q},\ddot{q})||\leq \vartheta$ presents the norm bounds. The above-mentioned expressions can be described by the following equation [33]:

(11) $$\{\begin{array}{l} M_{X}=J^{-T}MJ^{-1}\\ C_{X}=J^{-T}(C-MJ^{-1}\dot{J})J^{-1}\\ G_{X}=J^{-T}G\\ F_{end}=J^{-T}\tau_{end}\\ F_{d}=J^{-T}\tau_{d} \end{array}$$

### 3.2. Unified Dynamics Model Combined with the Target Object

**Figure 2:**
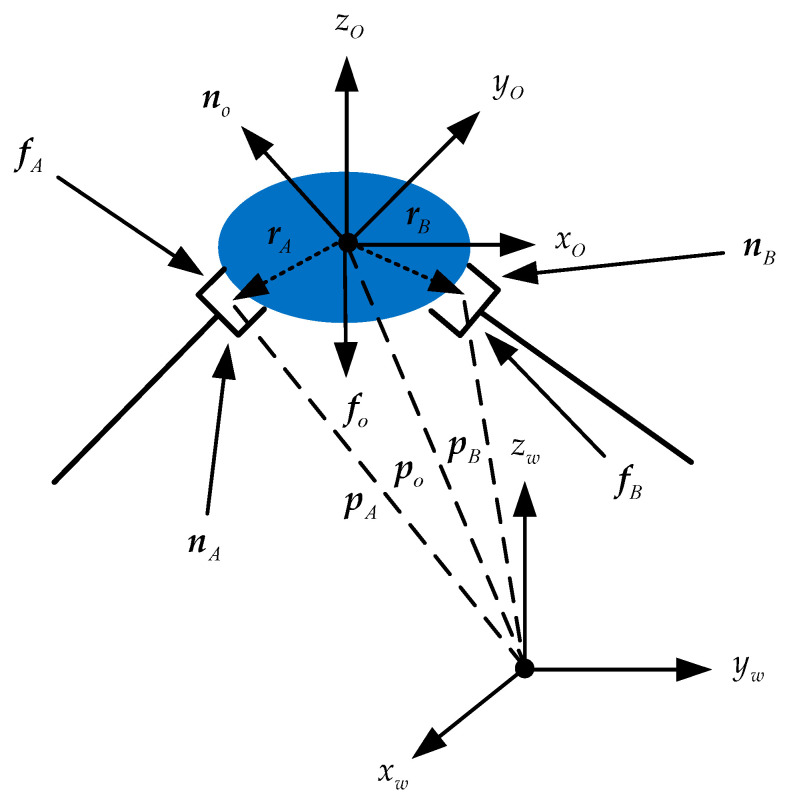
The diagram of the force of the object grasped by the dual-arm robots.

When the dual-arm end-effectors collaboratively grasp the object to move in the Cartesian space, the dual-arm robots and the held object form a closed chain constraint structure. The motion of the predetermined trajectory and the constraint control of the internal force are realized by the generalized driving force exerted by the end-effectors on the target object. Let the generalized output driving forces at the end-effectors be expressed as $F_{A}={\left[f_{A}^{T},n_{A}^{T}\right]}^{T}$ and $F_{B}={\left[f_{B}^{T},n_{B}^{T}\right]}^{T}$, respectively, the resultant force of the object’s COM is expressed as $F_{o}={\left[f_{o}^{T},n_{o}^{T}\right]}^{T}$, $p_{o}$ represents the position vector of the object’s COM in the robot world frame, and $r_{A}$ and $r_{B}$ are the position vector of the end-effectors with regard to the object’s COM, respectively. Figure 2 illustrates the force diagram of the manipulated object.

According to Newton’s force balance equation and Euler’s moment balance equation (Newton–Euler Equation), the dynamics equation of the object’s COM can be written in the following form:

(12) $$\{\begin{array}{l} f_{o}=m_{o}a_{o}-m_{o}g\\ n_{o}=I_{o}{\dot{\omega }}_{o}+\omega_{o}\times I_{o}\omega_{o} \end{array}$$

where $m_{o}$ is the object mass, g is the acceleration vector of the gravitational force, $a_{o}$ is the acceleration vector of the object’s COM, $\omega_{o}$ and ${\dot{\omega }}_{o}$ represent the angular position vector and angular velocity vector of the object’s COM, respectively, and $I_{o}$ is the inertia tensor of the manipulated object.

The generalized output force at the robot and end-effectors acts on the object’s COM by the contact point to complete the motion planning task. The generalized output forces at the object’s COM from the dual-arm end-effectors are respectively expressed as:

(13) $$F_{oA}=\left[\begin{array}{c} f_{A}\\ n_{A}+r_{A}\times f_{A} \end{array}\right]$$

(14) $$F_{oB}=\left[\begin{array}{c} f_{B}\\ n_{B}+r_{B}\times f_{B} \end{array}\right]$$

Combined with Equations (12)–(14), we obtain:

(15) $$F_{o}=F_{oA}+F_{oB}=G_{o}F_{e}$$

where $F_{e}={\left[F_{oA}^{T},F_{oB}^{T}\right]}^{T}$, and the matrix $G_{o}=\left[I_{6},I_{6}\right]\in R^{6\times 12}$ is the grasp matrix of the end-effectors acting on the object’s COM.

According to Equation (15), the end-effector output forces of the dual-arm robots acting on the object’s COM can be written:

(16) $$F_{e}=G_{o}^{+}F_{o}+(I_{12}-G_{o}^{+}G_{o})\lambda =F_{eE}+F_{eI}$$

where the first term $G_{o}^{+}F_{o}$ on the right side of Equation (16) represents the output force at the object’s COM by the robot end-effectors that drive the target object to move along the desired kinematics trajectory in the Cartesian space. Grasp matrix $G_{o}$ is not a square matrix. Its generalized inverse matrix satisfies the equality $G_{o}^{+}=W^{-1}G_{o}^{T}{\left(G_{o}W^{-1}G_{o}^{T}\right)}^{-1}$. W is the positive definite weighted matrix that plays a significant role in the motion performance of the object. To avoid the squeeze damage of the target object from the external force, we can get a generalized inverse matrix: $G_{o}^{+}=\frac{1}{2}{\left[I_{6},I_{6}\right]}^{T}\in R^{12\times 6}$. The second term $(I_{12}-G_{o}^{+}G_{o})\lambda$ on the right side of Equation (16) represents the internal force at the object’s COM which does not affect the motion trajectory of the object. The internal force consists of some pressures, tensions, and moments, etc., and $\lambda ={\left[\lambda_{A}^{T},\lambda_{B}^{T}\right]}^{T}\in R^{12\times 1}$ is the column vector of any magnitude [30,34]. Forces $F_{eIA}$ and $F_{eIB}$ from this term $(I_{12}-G_{o}^{+}G_{o})\lambda ={\left[F_{eIA}^{T},F_{eIB}^{T}\right]}^{T}$ are the internal force generated by the manipulator A and manipulator B at the object’s COM, respectively, and the internal force is in the null space projection of the matrix $G_{o}$.

As depicted above, because the end-effector output forces acting on the object’s COM $F_{e}$ can be decomposed into two parts, $F_{eE}$ and $F_{eI}$, the internal force exists in the null space of $G_{o}$, external forces and internal forces can be decoupled into two parts.

To facilitate the transformation between homogeneous transformation matrices in different coordinate systems, it is assumed that the *x*-, *y*-, and *z*-axis coordinates of coordinate systems $\left\{O_{A}\right\}$, $\left\{O_{B}\right\}$, and $\left\{O_{O}\right\}$ are in the same direction so that the position vectors of the manipulator A, the manipulator B, and the object’s COM in the dual-arm coordinated system satisfy the following equalities:

(17) $$x=\left[\begin{array}{c} x_{A}\\ x_{B} \end{array}\right]=\left[\begin{array}{l} p_{A}\\ \omega_{A}\\ p_{B}\\ \omega_{B} \end{array}\right]=\left[\begin{array}{l} p_{o}+R_{o}r_{A}\\ \omega_{o}\\ p_{o}+R_{o}r_{B}\\ \omega_{o} \end{array}\right]$$

where $R_{o}$ is a rotation matrix of the object’s COM and is expressed as a constant matrix. The position vectors $r_{A}$ and $r_{B}$ are constant vectors. Equation (17) defines a set of kinematics constraint relationships on the position and orientation of the dual-arm robot end-effectors; thus, they are always accomplished during manipulator system’s motion. Then, the following equation can be obtained:

(18) $$\{\begin{array}{l} \dot{x}={\dot{X}}_{o}\\ \ddot{x}={\ddot{X}}_{o} \end{array}$$

where ${\dot{X}}_{o}={\left[{\dot{x}}_{o}^{T},{\dot{x}}_{o}^{T}\right]}^{T}$ and ${\dot{x}}_{o}={\left[{\dot{p}}_{o}^{T},{\dot{\omega }}_{o}^{T}\right]}^{T}$. With the ideal conditions, the object’s COM and the dual-arm end-effectors have the same velocity, acceleration, and fixed pose constraint relationships.

By substituting Equation (18) into Equation (10), replacing the reaction force $F_{end}$ with the internal force $F_{eI}$ of the object’s COM, and considering the constraint relationships of the pose, velocity, and acceleration between the object’s COM and robot end-effectors, the unified dynamics equation between the target object and the dual-arm robots can be obtained:

(19) $$M_{X}(q){\ddot{X}}_{o}+C_{X}(q,\dot{q}){\dot{X}}_{o}+G_{X}(q)+F_{eI}+F_{d}+\Phi (q,\dot{q},\ddot{q})=F$$

## 4. Coordinated Transport Operation Based on Modified Sliding Mode Impedance Controller

The robots and the external environments can be integrated into an unified system using the dual-arm cooperative impedance control algorithm, and the desired kinematics relationship of the target impedance can be established while the real-time position control and force control of the robots can be carried out [35,36]. The traditional sliding mode controller (SMC) with the signum function will have a sharp chattering phenomenon, which will aggravate the wear consumption between the joints of the manipulators and shorten the service life of the manipulators. In this paper, the dual-arm impedance control strategy combined with the improved sliding mode control algorithm is used to reduce the chattering phenomenon caused by the switch of the acceleration control input in the SMC, and the tracking control of the end-effector positions and internal forces are simultaneously completed. The impedance control can also ensure the compliance characteristic of the task space.

### 4.1. Cooperative Impedance Control Strategy for Dual-Arm

**Figure 3:**
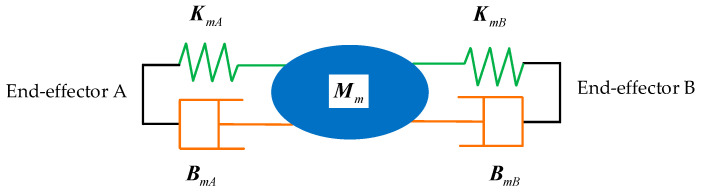
The diagram of impedance control structure.

When the dual-arm robots cooperatively carry a common rigid object to perform a trajectory planning task, the object and the two end-effectors form a complete closed constraint system. The end-effectors and rigid object constitute an impedance control structure to achieve the desired impedance control effect. The diagram of the impedance control structure of dual-arm robots is shown in Figure 3.

According to the unified dynamics model established by Equation (19), the impedance control equation in the Cartesian space for the target object can be obtained:

(20) $$M_{m}({\ddot{X}}_{od}-{\ddot{X}}_{o})+B_{m}({\dot{X}}_{od}-{\dot{X}}_{o})+K_{m}(X_{od}-X_{o})=F_{eI}+F_{d}$$

where $M_{m}\in R^{12\times 12}$, $B_{m}\in R^{12\times 12}$, and $K_{m}\in R^{12\times 12}$ represent the inertia, damping, and stiffness coefficient matrices of the grasped object, respectively, and $X_{od}$, ${\dot{X}}_{od}$, and ${\ddot{X}}_{od}$ denote the desired position, velocity, and acceleration vectors of the grasped object in the Cartesian space, respectively.

The manipulated object may drop out during the dual-arm robots’ coordinated transport operation. To prevent the mutation of the internal force of the manipulated object, the desired internal force $F_{eId}$ and the coefficient gain matrix $K_{eId}$ are introduced into the impedance control equation. Rearranging Equation (20), we obtain:

(21) $$M_{m}({\ddot{X}}_{od}-{\ddot{X}}_{o})+B_{m}({\dot{X}}_{od}-{\dot{X}}_{o})+K_{m}(X_{od}-X_{o})=K_{eId}(F_{eId}-F_{eI})+F_{d}$$

The expression form of the actual acceleration vector of the target object can be obtained from Equation (21)

(22) $${\ddot{X}}_{o}={\ddot{X}}_{od}+M_{m}^{-1}(B_{m}{\dot{\tilde{X}}}_{o}+K_{m}{\tilde{X}}_{o}-K_{eId}{\tilde{F}}_{eI}-F_{d})$$

where ${\tilde{X}}_{o}=X_{od}-X_{o}$, ${\dot{\tilde{X}}}_{o}={\dot{X}}_{od}-{\dot{X}}_{o}$, and ${\tilde{F}}_{eI}=F_{eId}-F_{eI}$. By combining Equation (7) with Equation (18), the desired angular acceleration ${\ddot{q}}_{d}$ of the inner ring of the dynamics control based on the joint space can be obtained:

(23) $$\begin{array}{cc} {\ddot{q}}_{d} & =J^{-1}(q)({\ddot{X}}_{o}-\dot{J}(q)\dot{q})\\ & =J^{-1}(q)({\ddot{X}}_{od}+M_{m}^{-1}(B_{m}{\dot{\tilde{X}}}_{o}+K_{m}{\tilde{X}}_{o}-K_{eId}{\tilde{F}}_{eI}-F_{d}))-\dot{J}(q)\dot{q}) \end{array}$$

The impedance control algorithm can dynamically adjust the trajectory tracking accuracy and force tracking accuracy of the manipulators, and the impedance model established by three impedance coefficient matrices can completely describe the dynamics characteristics of the dual-arm systems. The selection of diagonal elements of the coefficient matrices is crucial to the stability and position/force tracking effect of the dual-arm systems. Only by selecting reasonable impedance control parameters can we accomplish the coordinated handling and compliant control of the dual-arm manipulators.

### 4.2. Collaborative Impedance Algorithm Based on Modified Sliding Mode Controller

To improve the tracking accuracy of the impedance control algorithm, sliding mode control has been widely used in robot control systems with uncertain parameters and external disturbances due to its strong adaptive ability, robustness, and simple physical implementation. The robustness of the control system has the ability to keep certain performance unchanged under the external disturbance, and it plays an important role in the stable position/internal force tracking of the manipulator. A sliding mode control algorithm can be adopted to optimize the desired angular acceleration ${\ddot{q}}_{d}$ derived from the impedance control equation to achieve the precise position/force tracking results for the dual-arm robots.

The tracking error of the robot joint position is defined as:

(24) $$e(t)=q_{d}(t)-q(t)$$

Then, we consider the error function, i.e., the sliding surface s, such that:

(25) $$s=\dot{e}+\Lambda e$$

where Λ is a constant positive definite diagonal matrix.

Thus, the sliding mode impedance controller (SMIC) based on the signum function can be obtained, i.e., SMIC(sgn):

(26) $$\{\begin{array}{l} \tau =M{\ddot{q}}_{u}+C\dot{q}+G\\ {\ddot{q}}_{u}={\ddot{q}}_{d}+\Lambda \dot{e}+Ksgn(s) \end{array}$$

where ${\ddot{q}}_{u}$ is the angular acceleration control vector, K is the constant positive definite diagonal matrix used as the gain matrix of the switch function, and sgn(x) is the signum function given by:

(27) $$sgn(x)=\{\begin{array}{l} 1,\;\;\;\;\;x>0\\ 0,\;\;\;\;\;x=0\\ -1,\;\;x<0 \end{array}$$

**Figure 4:**
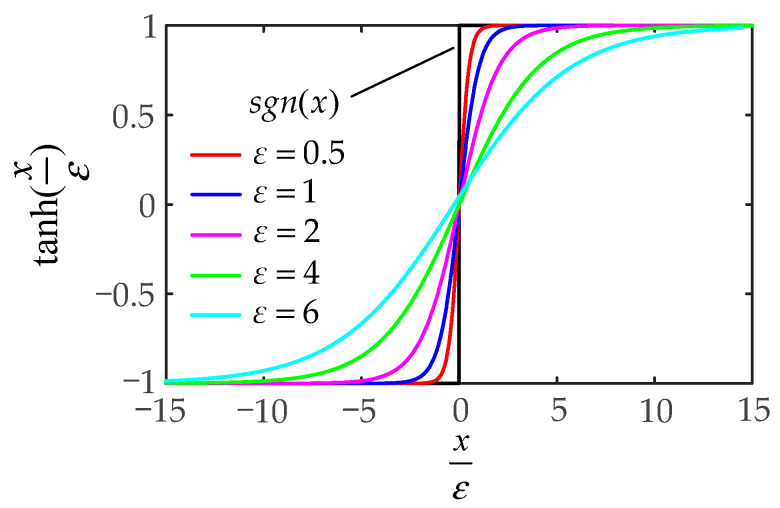
The images of the signum function sgn(x) and the hyperbolic tangent function $tanh(\frac{x}{\varepsilon })$.

The sgn(x) in the above controller will generate great instability of the input torques. To suppress the chattering caused by the signum function, the hyperbolic tangent function is used instead of the signum function, which can effectively reduce the chattering extent. The hyperbolic tangent function is defined as

(28) $$tanh(\frac{x}{\varepsilon })=\frac{e^{\frac{x}{\varepsilon }}-e^{-\frac{x}{\varepsilon }}}{e^{\frac{x}{\varepsilon }}+e^{-\frac{x}{\varepsilon }}}$$

where ε>0 is used to adjust the curvature of the hyperbolic tangent function. The ascending curve of the signum function sgn(x) and the hyperbolic tangent function $tanh(\frac{x}{\varepsilon })$ is shown in Figure 4.

**Figure 5:**
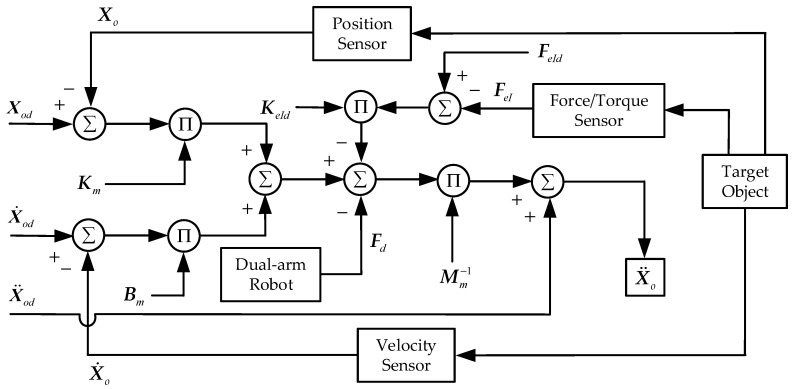
The motion model block diagram based on impedance control strategy.

**Figure 6:**
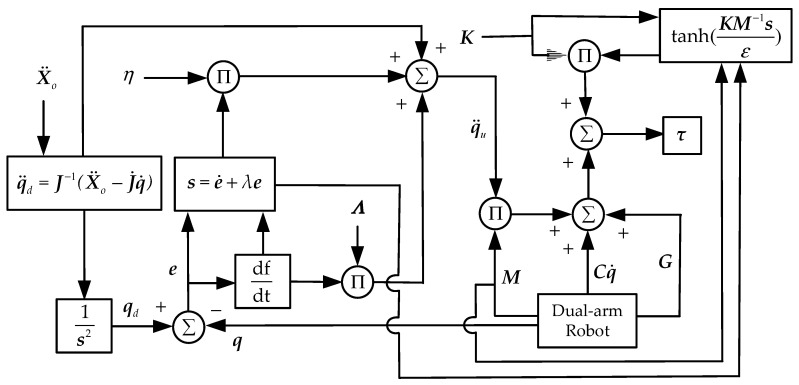
The torque output block diagram based on modified sliding mode control strategy.

The SMIC(sgn) can effectively control the inertia term but lacks the error compensation term of the Coriolis and centrifugal force matrix and the gravitational force vector. The modified sliding mode impedance controller based on the hyperbolic tangent function, i.e., MSMIC(tanh), is given by:

(29) $$\{\begin{array}{l} \tau =M{\ddot{q}}_{u}+C\dot{q}+G+Ktanh(\frac{KM^{-1}s}{\varepsilon })\\ {\ddot{q}}_{u}={\ddot{q}}_{d}+\Lambda \dot{e}+\eta s\\ {\ddot{q}}_{d}=J^{-1}(q)({\ddot{X}}_{o}-\dot{J}(q)\dot{q})\\ {\ddot{X}}_{o}={\ddot{X}}_{od}+M_{m}^{-1}(B_{m}{\dot{\tilde{X}}}_{o}+K_{m}{\tilde{X}}_{o}-K_{eId}{\tilde{F}}_{eI}-F_{d}) \end{array}$$

where $K=\mathrm{diag}\{k_{1},k_{2},\ldots ,k_{i},\ldots ,k_{12}\}$ presents the controller gain matrix, $k_{i}>0(i=1,2,\ldots ,12)$, and the term $Ktanh(\frac{KM^{-1}s}{\varepsilon })$ is used to compensate the control torque and improve the tracking accuracy of the control system. The motion model block diagram based on impedance control strategy is shown in Figure 5 and the torque output block diagram based on modified sliding mode control strategy is shown in Figure 6.

### 4.3. Analysis of Stability and Convergence

The MSMIC(tanh) controller combines the dual-arm impedance control algorithm with the modified sliding mode control algorithm. To prove the stability and convergence of the designed controller, the following two theorems about the hyperbolic tangent function are given [37,38]:

**Theorem** **1.** *If*∀x∈R*, as a given number, then the following inequality holds:*

(30) $$|x|-xtanh(\frac{x}{\varepsilon })\leq \rho \varepsilon$$

*where*ε>0*is a numerator coefficient that affects the curvature of the hyperbolic tangent function*$tanh(\frac{x}{\varepsilon })$*,*ρ=0.2785*is a constant term, and*ρε*is the upper limit of*$xtanh(\frac{x}{\varepsilon })$.

**Theorem** **2.** *Set* h,N: [0,∞)∈R*, the inequality equation satisfies*$\dot{N}\leq -\beta N+h$*, and its solution can be obtained:*

(31) $$N(t)\leq e^{-\beta (t-t_{0})}N(t_{0})+\int_{t_{0}}^{t}e^{-\beta (t-\sigma )}h(\sigma )d\sigma$$

*where*β∈R*and*$\forall t\geq t_{0}\geq 0$.

The stability and convergence analysis of the proposed controller is carried out below. Combining Equations (8), (25), and (29) when considering the two theorems yields:

(32) $$\begin{array}{cc} \dot{s} & =\ddot{e}+\Lambda \dot{e}\\ & ={\ddot{q}}_{d}-\ddot{q}+\Lambda \dot{e}\\ & ={\ddot{q}}_{d}-M^{-1}(\tau -\tau_{d}-\tau_{end}-G-C\dot{q})+\Lambda \dot{e}\\ & ={\ddot{q}}_{d}-M^{-1}(M({\ddot{q}}_{d}+\Lambda \dot{e}+\eta s)+C\dot{q}+G+Ktanh(\frac{KM^{-1}s}{\varepsilon })-\tau_{d}-\tau_{end}-G-C\dot{q})+\Lambda \dot{e}\\ & =-\eta s-M^{-1}Ktanh(\frac{KM^{-1}s}{\varepsilon })+M^{-1}\tau * \end{array}$$

where $\tau *=\tau_{d}+\tau_{end}$, $\tau *={\left[\tau_{1}*,\tau_{2}*,\ldots \tau_{i}*,\ldots ,\tau_{12}*\right]}^{T}$, i=1,2,…,12.

Define the Lyapunov function V as:

(33) $$V=\frac{1}{2}s^{T}s$$

Taking the time derivative of the Lyapunov function V, we can obtain Equation (34):

(34) $$\begin{array}{cc} \dot{V} & =\frac{1}{2}s^{T}\dot{s}+\frac{1}{2}{\dot{s}}^{T}s=s^{T}\dot{s}\\ & =s^{T}(-\eta s-M^{-1}Ktanh(\frac{KM^{-1}s}{\varepsilon })+M^{-1}\tau *)\\ & =-\eta s^{T}s-s^{T}M^{-1}Ktanh(\frac{KM^{-1}s}{\varepsilon })+s^{T}M^{-1}\tau * \end{array}$$

Let us consider Theorem 1 such that:

(35) $$-s^{T}M^{-1}K\tanh(\frac{KM^{-1}s}{\varepsilon })\leq -\left\|s^{T}M^{-1}K\right\|+\rho \varepsilon$$

where ‖f(⋅)‖ represents the least two norms of the vector f(⋅).

According to Equations (33) and (35), Equation (34) can be reformulated as:

(36) $$\begin{array}{cc} \dot{V} & =-\eta s^{T}s-s^{T}M^{-1}Ktanh(\frac{KM^{-1}s}{\varepsilon })+s^{T}M^{-1}\tau *\\ & \leq -\eta s^{T}s-\left\|s^{T}M^{-1}K\right\|+\rho \varepsilon +s^{T}M^{-1}\tau *\\ & \leq -\eta s^{T}s+\rho \varepsilon =-2\eta V+a \end{array}$$

where the inequality term satisfies $-\left\|s^{T}M^{-1}K\right\|+s^{T}M^{-1}\tau *\leq 0$ and the constant term satisfies equality a=ρε.

The inequality $\dot{V}\leq -2\eta V+a$ can be obtained. Considering Theorem 2, we get:

(37) $$\begin{array}{cc} V(t) & \leq e^{-2\eta (t-t_{0})}V(t_{0})+ae^{-2\eta t}\int_{t_{0}}^{t}e^{2\eta \sigma }d\sigma \\ & =e^{-2\eta (t-t_{0})}V(t_{0})+\frac{ae^{-2\eta t}}{2\eta }(e^{2\eta t}-e^{2\eta t_{0}})\\ & =e^{-2\eta (t-t_{0})}V(t_{0})+\frac{a}{2\eta }(1-e^{-2\eta (t-t_{0})})\\ & =e^{-2\eta (t-t_{0})}V(t_{0})+\frac{\rho \varepsilon }{2\eta }(1-e^{-2\eta (t-t_{0})}) \end{array}$$

Equation (37) shows that the inequality $\underset{t\to \infty }{\lim}V(t)\leq \frac{\rho \varepsilon }{2\eta }$, that the tracking error e and its derivative $\dot{e}$ converge asymptotically, and that the convergence effect of the Lyapunov function depends on ε and η. It can be seen that the designed MSMIC(tanh) is stable and convergent.

## 5. Simulation Results and Analysis

**Figure 7:**
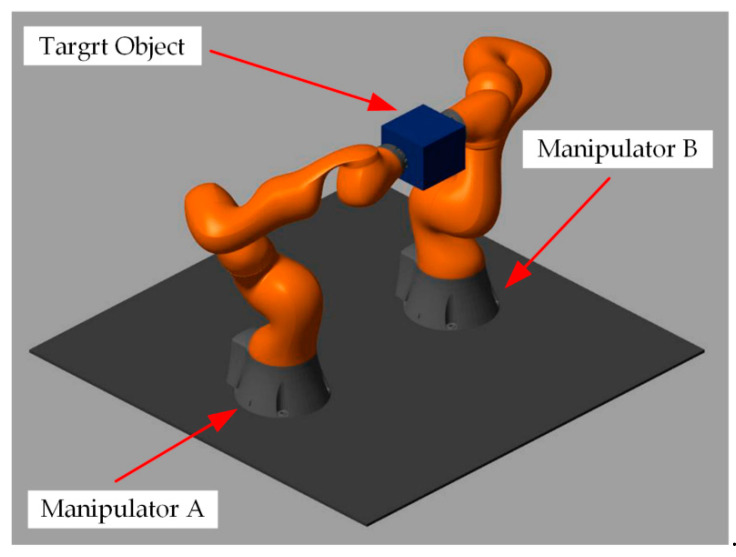
The initial state of the coordinated operation system for the dual-arm robots.

This section mainly verifies the feasibility and effectiveness of the proposed controller based on the modified sliding mode impedance scheme. MATLAB R2020a and SIMULINK soft are used as the simulation experiment platform, combined with the Robotics System Toolbox and SimMechanics plugin (see Figure 7). Two Kuka iiwa14 manipulator models are used to establish the dual-arm robot systems, and the dual-arm coordinated transport experiment is tested in this simulation system. The robot is a redundant manipulator composed of seven revolute joints R⊥R⊥R⊥R⊥R⊥R⊥R. The redundant degrees of freedom can make the manipulator have more joint space configurations in the process of motion to increase the flexibility and diversity of the trajectory planning. The mass of each link of the manipulator is shown in Table 1 and the DH parameters are shown in Table 2. At the same time, the simulation experiment of force tracking with the impedance-free control (IFC) algorithm and the simulation experiment of SMIC(sgn) based on the signum function are also conducted in this section as the contrast group to highlight the superiority and advancement of the proposed control algorithm for position/force tracking effect.

The object grasped by the dual-arm robots is a cube with uniform mass distribution with the length of the side $l_{o}=0.15m$, mass $m_{o}=3.375\mathrm{kg}$, and gravitational acceleration vector $g=[0,0,-9.8m/s^{2}]$. The initial position of the object’s COM in the world frame is (0.2m,0,0.8m). The position of the base origin of the manipulator A in the world frame is (0,−0.3m,0), and the position of the base origin of the manipulator B in the world frame is (0,0.3m,0). The origin positions of the two end-effectors are (0.2m,−0.075m,0.8m) and (0.2m,0.075m,0.8m), respectively. Then, the initial positions of the corresponding joint angles (in radian system) are obtained:

$$\{\begin{array}{c} q_{start1}={\left[1.61,-0.61,-0.56,-1.85,-2.28,-0.56,0.46\right]}^{T}\\ q_{start2}={\left[-1.61,-0.61,0.56,-1.85,2.28,-0.56,-0.46\right]}^{T} \end{array}$$

The target object is planned by using the circle trajectory in Cartesian space:

(38) $$\{\begin{array}{l} x_{o}=rcos\theta (t)\\ y_{o}=rsin\theta (t)\\ z_{o}=0.8 \end{array}$$

where θ(t) is a quintic polynomial interpolation function about time t, which is used to produce the desired trajectory of the object with the radius r=0.2 m. Assuming the total system simulation time is T=10 s and the sampling period of the SIMULINK solver is dt=0.01 s:

The relevant parameters of the proposed controller are selected as follows: the constant η=2, and the denominator coefficient of the hyperbolic tangent function ε=10. The gain matrix of the sliding mode controller and the gain matrix of the sliding function are chosen as:

$$\{\begin{array}{l} K=\mathrm{diag}[1,1,1,1,1,1,1,1,1,1,1,1,1,1]\\ \Lambda =\mathrm{diag}[140,140,140,140,140,140,140,140,140,140,140,140,140,140] \end{array}$$

The desired internal force of the object is 15N in the positive direction of the *y*-axis of the world frame. The desired internal force gain matrix is chosen as $K_{eId}=\mathrm{diag}[0,5,0,0,0,0,0,5,0,0,0,0]$. The inertia matrix, damping matrix, and stiffness matrix of the manipulated object are chosen as:

$$\{\begin{array}{l} M_{m}=\mathrm{diag}[10,10,10,10,10,10,10,10,10,10,10,10]\\ B_{m}=\mathrm{diag}[100,100,100,100,100,100,100,100,100,100,100,100]\\ K_{m}=\mathrm{diag}[20,20,20,20,20,20,20,20,20,20,20,20] \end{array}$$

**Figure 8:**
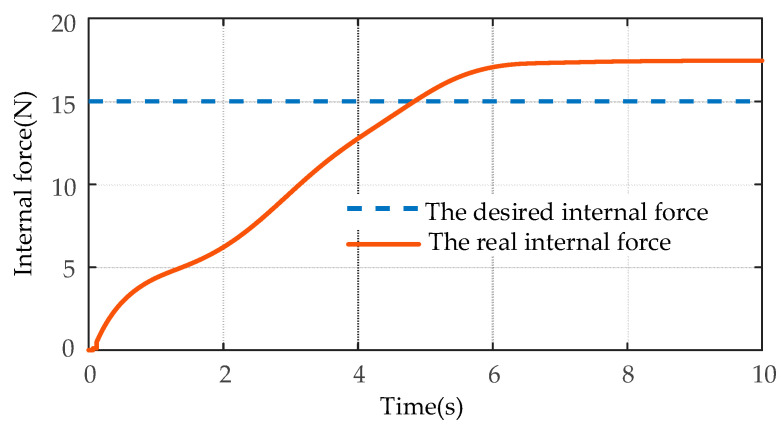
The simulation results of the internal force tracking based on the IFC algorithm.

The simulation results of the internal force tracking based on the IFC algorithm are shown in Figure 8. As shown in Figure 8, the IFC algorithm cannot track the desired internal force of the *y*-axis, which indicates that the sliding mode controller that does not use the impedance control algorithm cannot achieve the coordinated transport of the object target.

**Figure 9:**
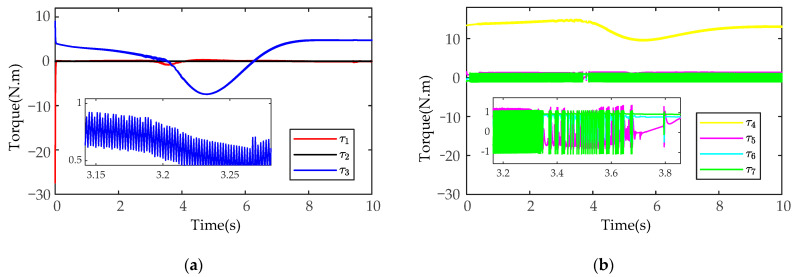
The simulation results of control torque based on the SMIC(sgn) algorithm: (**a**) the control torque for the joints 1-2-3; (**b**) the control torque for the joints 4-5-6-7.

The simulation results of control torque based on the SMIC(sgn) algorithm are shown in Figure 9 (only the control torque of the manipulator A is analyzed). As shown in Figure 9, the joint torque chattering of the SMIC(sgn) algorithm is larger, and there is an obvious periodic oscillation phenomenon. In a practical manipulator operation task, the periodic chattering will cause great wear and tear to the joints and links of manipulators.

**Figure 10:**
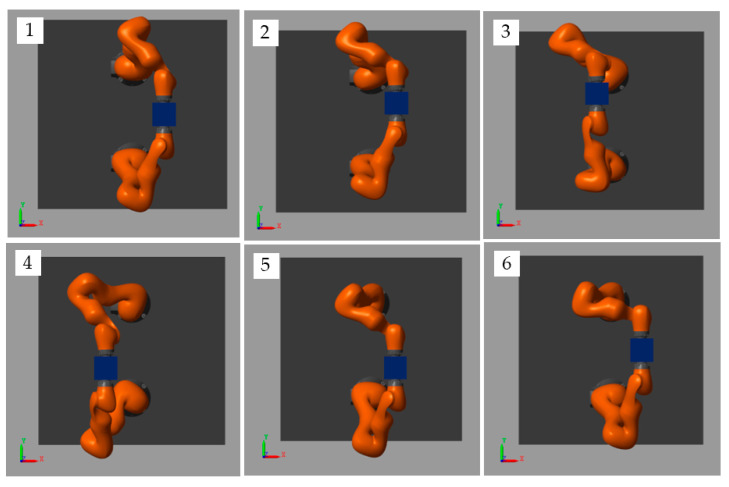
Snapshots of this simulation for target object along a circle trajectory.

The simulation snapshots of the MSMIC(tanh) algorithm proposed in this paper are shown in Figure 10.

**Figure 11:**
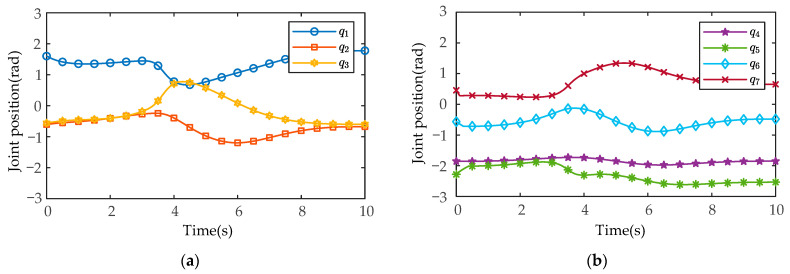
The measured results of the actual joint positions based on the MSMIC(tanh) algorithm for the manipulator A: (**a**) the actual angle positions for the joints 1-2-3; (**b**) the actual angle positions for the joints 4-5-6-7.

**Figure 12:**
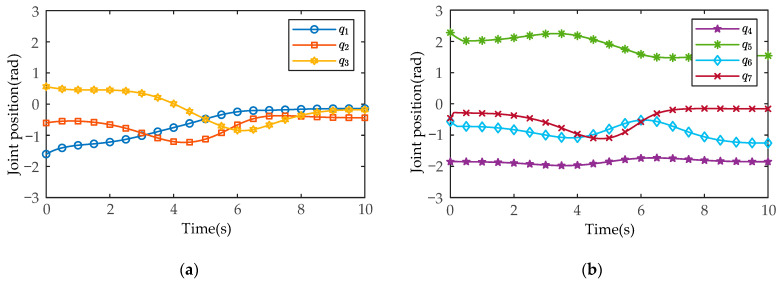
The measured results of the actual joint positions based on the MSMIC(tanh) algorithm for the manipulator B: (**a**) the actual angle positions for the joints 1-2-3; (**b**) the actual angle positions for the joints 4-5-6-7.

**Figure 13:**
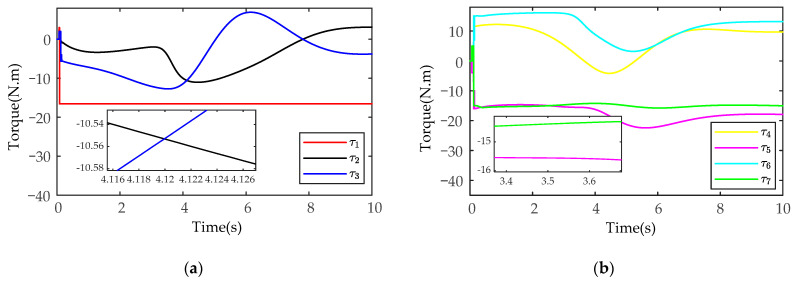
The simulation results of the joint control torques based on the MSMIC(tanh) algorithm for the manipulator A: (**a**) the control torques for the joints 1-2-3; (**b**) the control torques for the joints 4-5-6-7.

**Figure 14:**
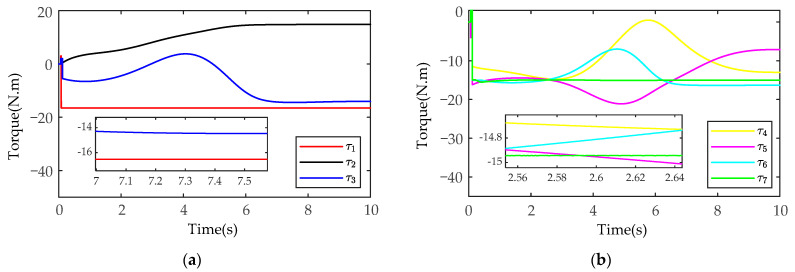
The simulation results of the joint control torques based on the MSMIC(tanh) algorithm for the manipulator B: (**a**) the control torques for the joints 1-2-3; (**b**) the control torques for the joints 4-5-6-7.

After MATLAB and SIMULINK system simulation, the actual joint positions of the manipulators measured by the joint position sensors in the SimMechanics plugin are shown in Figure 11 and Figure 12 (manipulator A and manipulator B), and the joint control torques are shown in Figure 13 and Figure 14.

As shown in Figure 11 and Figure 12, in the process of coordinated transport object by the dual-arm robots, the joint angles run smoothly without chattering and mutation. At the same time, the joint positions do not exceed the upper and lower angle limit. As shown in Figure 13 and Figure 14, the output torques of the manipulators are only transient unstable in the initial run moment, and the manipulators can generate the required stabilizing control torques at about t=0.15s. Thereafter, there is no periodic chattering phenomenon existing in the traditional sliding mode controller, which indicates the effectiveness of the proposed MSMIC(tanh) algorithm in suppressing torque chattering (it can be seen more clearly and carefully from the torque zoom diagram). It is also shown that the selection of the impedance parameter matrices, the sliding mode gain matrix, and the hyperbolic tangent function denominator coefficient is reasonable, which play a key role in generating stable angle positions and control torques of dual-arm manipulators.

**Figure 15:**
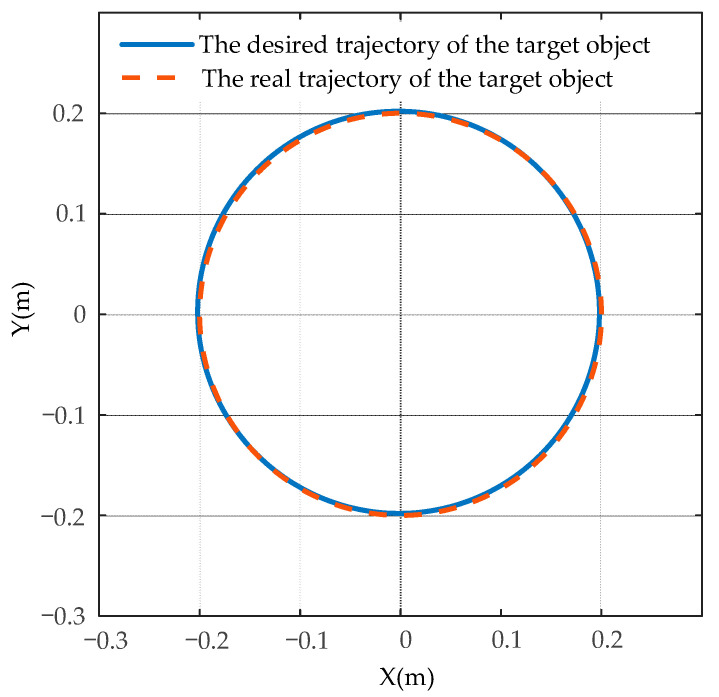
The comparison results of the desired and the actual trajectory of the target object using the MSMIC(tanh) algorithm.

**Figure 16:**
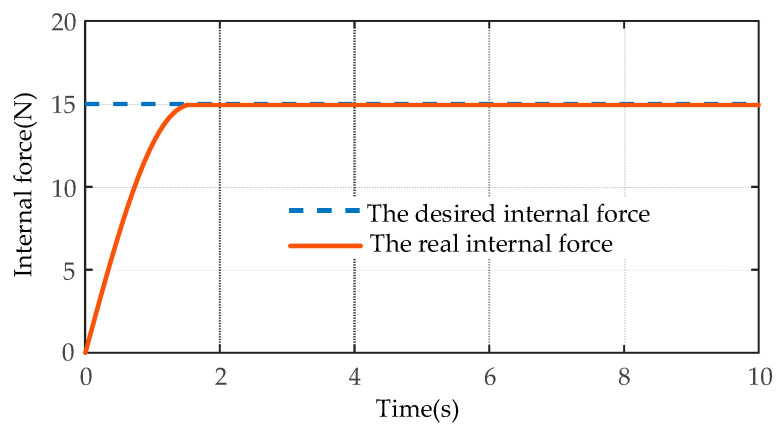
The simulation comparison results of the internal force tracking based on the MSMIC(tanh) algorithm.

The comparison results of the desired circle trajectory and the actual trajectory of the target object using the MSMIC(tanh) algorithm are shown in Figure 15. The tracking effects of the desired and the actual internal forces on the *y*-axis are shown in Figure 16.

As shown in Figure 15, the dual-arm robot systems that are driven by the control torques of the MSMIC(tanh) algorithm proposed in this paper can grasp collaboratively the object along the circle trajectory and generate the high-accuracy trajectory tracking effect. As shown in Figure 16, the force tracking control algorithm can track the desired internal force at about *t* = 1.74 s, and then remains in a relatively stable force tracking state until the end of the operation task. Therefore, it can be concluded that the end-effectors of the dual-arm can accomplish the cooperative transport task of the target object using the MSMIC(tanh) controller proposed in this paper. At the same time, the circle trajectory tracking and internal force tracking for the object with high precision are well implemented.

Next, the disturbance simulation tests of the designed controller are carried out. Taking the second joint of manipulator A, which is obviously affected by gravity and terminal disturbance, as an example, the expected joint position is designed as a sine function $q_{2}=sin(t)$, and the expected angular velocity is designed as a cosine function ${\dot{q}}_{2}=cos(t)$. A time-varying disturbance force of the external environment is added to the end-effector of the manipulator A along the *z*-axis, and the variation rule of the disturbance force is $f_{d}=10sin(t)N$. The simulation experiment results of angular position and angular velocity of SMIC(sgn) and MSMIC(tanh) controllers are compared under the time-varying disturbance force.

**Figure 17:**
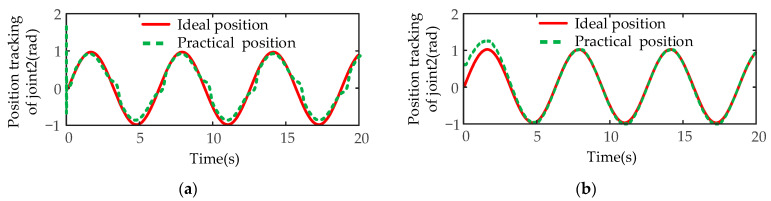
The position tracking results of the disturbance simulation test for the second joint of manipulator A: (**a**) the position tracking results for the SMIC(sgn) controller; (**b**) the position tracking results for the MSMIC(tanh) controller.

The position tracking results of the two controllers are shown in Figure 17. Figure 17a shows that the trajectory tracking result of the traditional sliding mode controller based on the signum function is poor due to the influence of time-varying disturbance force. It can be seen from Figure 17b that the MSMIC(tanh) controller has a relatively high-precision trajectory tracking, and the joint position error is small after 2.5 s. The modified controller proposed completes the tracking task of the desired trajectory.

**Figure 18:**
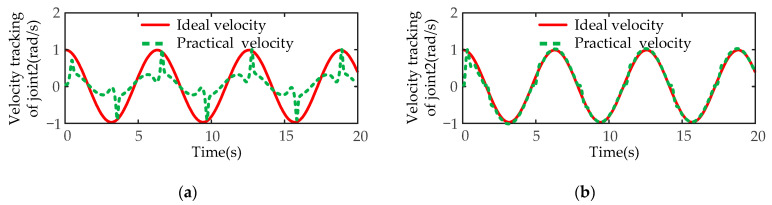
The velocity tracking results of the disturbance simulation test for the second joint of manipulator A: (**a**) the velocity tracking results for the SMIC(sgn) controller; (**b**) the velocity tracking results for the MSMIC(tanh) controller.

The tracking results of angular velocity are shown in Figure 18. Figure 18a shows that the SMIC(sgn) controller is difficult to track the expected angular velocity, and the velocity error is relatively big, so the conventional algorithm almost fails. On the contrary, the MSMIC(tanh) controller successfully realizes the tracking task of the angular velocity (see Figure 18b. The joint velocity error is large only at the moment when the disturbance force changes most dramatically. The MSMIC(tanh) controller can achieve a better velocity tracking effect.

**Figure 19:**
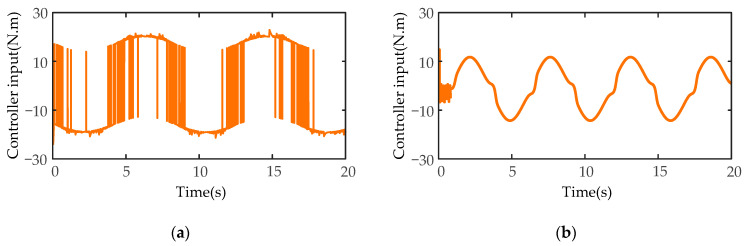
The controller torque results of the disturbance simulation test for the second joint of manipulator A: (**a**) the controller torque results for the SMIC(sgn) controller; (**b**) the controller torque results for the MSMIC(tanh) controller.

Finally, the controller torque comparison results are shown in Figure 19. Since the SMIC(sgn) controller has a signum function as a switch function, the input torque has a large chattering, the maximum chattering value is more than 40 N·m (see Figure 19a), which will cause wear damage to the joints of the manipulator. However, the MSMIC(tanh) controller obviously reduces the moment chattering phenomenon (See Figure 19b. Compared with the signum function, the hyperbolic tangent function can effectively avoid the chattering caused by the unsmooth switch function at the boundary layer and enhance the robustness and stability of the robot control system.

## 6. Discussion

In this paper, considering the task of cooperatively carrying the object with two manipulators, a coordinated control strategy for dual-arm robots based on modified sliding mode impedance controller is proposed. The combinative kinematics model of dual-arm robots and the unified dynamics equation combining dual-arm robots with target object are established, the modified sliding mode impedance controller is designed, the stability and convergence of the control system are analyzed, as well as the dual-arm robot coordinated handling simulation experiment in MATLAB/SIMULINK is finished. Simulation results show that compared with the previous control strategy, the proposed controller has reduced chattering amplitude and possessed faster response speed and steady-state performance. At the same time, the dual-arm robots can complete the high-precision trajectory tracking control and can meet the requirements of the desired contact force between the dual-arm end-effectors and the manipulated object and can finally achieve the coordinated carrying task for manipulators. The modified control strategy can compensate model errors term and uncertainty term, reduce the steady-state error of the end-effector trajectory tracking, improve the robustness and adaptability of the control system, and make the system have good dynamic and steady-state performance. Although the control strategy cannot solve all the dual-arm position/force coupling tasks, it provides a feasible solution in the coordinated handling operation task and has certain significance for the popularization of dual-arm robot compliant control of the object.

Dual-arm robots have attracted the attention of numerous researchers because of their cooperative and mutual assistance merits. A dual-arm robot can complete the coordinated handling operation task that cannot be completed with a single manipulator. In scenes such as freight docks and large logistics warehouses, dual-arm robots can be used for multiple procedures such as assembly, disassembly, sorting, handling, and packaging. A single manipulator is not up to the simple carrying task [39]. In this case, a dual-arm robot is needed to replace human labor and help enterprises save unnecessary costs [40]. What is more noteworthy is that the dual-arm robot control strategy in this paper can be applied toward teaching by demonstration for robot-assisted minimally invasive surgery [41]. We will also cooperate with robot enterprises. The designed controller is applied to the actual production line of industrial products. At the same time, we will further accurately establish the dynamics model of the dual-arm robot, perform the task of coordinated handling object in an unstructured environment, and consider the null space obstacle avoidance and end-effector obstacle avoidance tasks based on the RBF neural network momentum observer, etc.

## 7. Conclusions

To solve the problems of internal force instability, trajectory tracking of low precision, and joint torque chattering in the process of the dual-arm handling the target object, a modified sliding mode impedance controller based on the hyperbolic tangent function is proposed. Therefore, the combinative kinematics equation of the robot model and the unified dynamics equation with the held object are established. The coefficient gain matrix $K_{eId}$ is introduced into the impedance control equation to prevent the mutation of the internal force of the target object. The MSMIC(tanh) is designed by combining the impedance controller with the modified sliding mode controller to compensate the errors of modeling parameters and external disturbances of the dual-arm cooperative manipulators. The stability and convergence of the control algorithm are proved by Lyapunov function.

Finally, the simulation experiment results of IFC, SMIC(sgn), and MSMIC(tanh) of the coordinated handling object for the dual-arm robots are conducted in the MATLAB and SIMULINK system. Simulation results show that, compared with IFC and SMIC(sgn), the developed MSMIC(tanh) can output the required internal forces stably and achieve a high-precision trajectory tracking effect, solving the periodic torque and joint chattering problems existing in the traditional sliding mode control algorithm. The method is supported by theoretical analysis and simulation results. Time-varying disturbance simulation results show that the proposed MSMIC(tanh) controller can better achieve accurate approximation of the external environment uncertainty factors, effectively suppress the adverse effects of external uncertainties, and improve the position/velocity control accuracy of the manipulator.

## Figures and Tables

**Table 1:** The link mass of Kuka iiwa14 manipulator.

Link	Link1	Link2	Link3	Link4	Link5	Link6	Link7
Mass/(kg)	4	4	3	2.7	1.7	1.8	0.3

**Table 2:** The D-H parameters of Kuka iiwa14 manipulator.

Theta	d/(m)	a/(m)	Alpha/(rad)
$q_{1}$	0.36	0	−π/2
$q_{2}$	0	0	π/2
$q_{3}$	0.42	0	π/2
$q_{4}$	0	0	−π/2
$q_{5}$	0.40	0	−π/2
$q_{6}$	0	0	π/2
$q_{7}$	0.126	0	0

## Data Availability

Data sharing is not applicable to this article.
